# Lessons learned spanning 17 years of experience with three consecutive nationwide competency based medical education training plans

**DOI:** 10.3389/fmed.2024.1339857

**Published:** 2024-02-22

**Authors:** Merel H. de Heer, Erik W. Driessen, Pim W. Teunissen, Fedde Scheele

**Affiliations:** ^1^Amsterdam UMC Location Vrije Universiteit Amsterdam, Research in Education, Amsterdam, Netherlands; ^2^School of Health Professions Education (SHE), Faculty of Health Medicine and Life Sciences (FHML), Maastricht University, Maastricht, Netherlands; ^3^Department of Obstetrics and Gynecology, Maastricht University Medical Center (MUMC+), Maastricht, Netherlands; ^4^Athena Institute, Faculty of Science, VU, Amsterdam, Netherlands

**Keywords:** competency based medical education, postgraduate education, training plan, design, development, experience, implementation

## Abstract

**Introduction:**

Curricula for postgraduate medical education have transformed since the introduction of competency based medical education (CBME). Postgraduate training plans offer broader training with different competencies and an outcome-based approach, in addition to the medical technical aspects of training. However, CBME also has its challenges. Over the past years, critical views have been shared on the potential drawbacks of CBME, such as assessment burden and conflicts with practicality in the workplace. Recent studies identified a need for a better understanding of how the evolving concept of CBME has been translated to curriculum design and implemented in the practice of postgraduate training. The aim of this study was to describe the development of CBME translations to curriculum design, based on three consecutive postgraduate training programs spanning 17 years.

**Method:**

We performed a document analysis of three consecutive Dutch gynecology and obstetrics training plans that were implemented in 2005, 2013, and 2021. We used template analysis to identify changes over time.

**Results:**

Over time, CBME-based curriculum design changed in several domains. Assessment changed from a model with a focus on summative decision to one with an emphasis on formative, low-stakes assessments aimed at supporting learning. The training plans evolved in parallel to evolving educational insights, e.g., by placing increasing emphasis on personal development. The curricula focused on a competency-based concept by introducing training modules and personalized authorization based on feedback rather than on a set duration of internships. There was increasing freedom in personalized training trajectories in the training plans, together with increasing trust towards the resident.

**Conclusion:**

The way CBME was translated into training plans has evolved in the course of 17 years of experience with CMBE-based education. The main areas of change were the structure of the training plans, which became increasingly open, the degree to which learning outcomes were mandatory or not, and the way these outcomes were assessed.

## 1 Introduction

Competency based medical education (CBME) is an outcome based approach to education. The definition of CBME is highly variable in the literature (1). Frank et al. propose the following definition: CBME is an outcomes-based approach to the design, implementation, assessment, and evaluation of medical education programs, using an organizing framework of competencies (2). The outcome based approach is well aligned with workplace-based learning, such as postgraduate medical education, since all residents need to have a similar base of competence, mostly regarding medical accountability and patient safety. Already, in 1978 the WHO promoted the wide use of competency-based models in medical education (3). The popularity of CBME has increased ever since, as can be seen by increased implementation in postgraduate curricula (4) and the use of competency frameworks globally, such as the CanMEDS framework and ACGME Milestones (5, 6).

While the introduction of CBME has transformed training plans for postgraduate medical education, clinicians, educationalists and curriculum designers recognize the need for insight in how to use CBME (7, 8). There are few publications that share experiences and lessons learned from working with a CBME-based postgraduate training plan (9, 10). In this paper, we explore how the concept of CBME has evolved in training plans of postgraduate education for obstetrics and gynecology. We aim to identify lessons learned from 17 years of experience with CBME-based curriculum design in Dutch obstetrics and gynecology postgraduate education.

CBME has several characteristics, but the three core aspects of most CBME-based curricula are an outcome-based approach, workplace-based assessment and time-variable learning (7, 11–13). The outcome-based approach entails the translation of competency descriptions to practice in training plans. Workplace-based assessment covers how the residents’ progress towards their intended outcomes is monitored and assessed (14). Competency-based time-variable learning focuses on attained competencies, independent of the time needed to achieve those competencies (15).

Although CBME has been implemented worldwide and is a popular educational model for medical education, the literature describes several challenges related to CBME in postgraduate education (8, 14, 16–22). The challenges mentioned in literature can be categorized in three main themes, which are elaborated below: *the practicality of CBME* (20–22), the *assessment burden* (8, 23) and also the *developments in the work field* (18, 24–26). *The practicality of CBME* covers the way the educational concept is used and implemented. Implementation in an existing health system is challenging (2, 27). The challenge is often debated as a balance between strict implementation and sufficient flexibility to ensure that the concept works in a local context. Strict implementation of CBME with a compulsory and fixed curriculum can serve the intended goal of CBME, but this compulsory and fixed curriculum appears to be not realistic in practice and not in line with implementation literature (7, 21, 28, 29). CBME can come across as rigid or strict (7, 21, 28). In addition, several authors described *the assessment burden* as a mental burden on residents due to the high frequency of observations and assessments. At the same time, the assessment burden is also described as a time-consuming practical burden for teachers because of the frequent moments of direct observation and the cumbersome administration of assessment outcomes (8, 23). Challenges related to *the developments in the work field* entail several aspects: training plans remain in need of change due to ongoing developments in health care systems, such as shared decision-making (18), but also due to educational insights and innovations resulting from ongoing research on the educational and clinical care outcomes of CBME (26, 30). Examples of those educational insights could be the growth mindset (ability can increase by focus, persistence and coaching) and mastery goal orientation (development of effective intrapersonal learning strategies for current-and lifelong learning). These educational approaches align with the tenets of CBME well, but also focus on competences reaching beyond the medical training itself (25, 26). Although it can be challenging to adjust set competencies and training plans, CBME should be continuously refined and informed by ongoing innovation and developments (18, 25).

CBME was first implemented in the Dutch obstetrics and gynecology training plan 17 years ago. In 2005 the first competency-based O&G training plan was launched, followed by a revision in 2013, and the most recent training plan revision for O&G in the Netherlands took place in 2021. Over those years, the design of the training plans evolved to cope with the previously mentioned challenges. The aim of this study was to describe the development of CBME translations to curriculum design, based on three consecutive postgraduate training plans spanning 17 years. By exploring how CBME has been used in successive O&G training plans, we aim to elucidate how CBME-based curriculum design has evolved and which lessons curriculum designers have learned. The outcomes of this study can be used to inform the development of future postgraduate training plans.

## 2 Methods

We conducted a qualitative document analysis, using three national Dutch obstetrics and gynecology (O&G) training plans spanning 17 years. We used template analysis, which is a form of thematic analysis (31). Thematic analysis is a method for analyzing qualitative data that allows for the identification of repeated patterns or themes across the dataset (32). Template analysis offers a description of a number of practical steps to be undertaken that starts with the description of with *a-priori* themes derived from pre-existing knowledge in literature (31).

### 2.1 Setting and data

This study was set in the Netherlands, where the O&G training plan covers 6 years of training. At least 2 years of the training take place in an academic hospital, which is the main base for the specialty training, and at least 2 years take place in a general hospital within the same training region. In total, there are 8 academic hospitals, which form the nucleus of a training region, and 48 general hospitals that participate in the postgraduate education of O&G residents, divided over the eight training regions.

O&G has a national training plan as well as local training plans based on competency-based medical education. The national training plan describes the content and structure of assessment, the intended learning outcomes, quality assurance, and implementation strategies and provides guidelines for local training regions and hospitals. The national training plan also provides a theoretical background of the developments in the medical field and in educational concepts that are of influence on training plans. Local training plans effectuate the national training plan in the local context of the training hospital. For this study, we analyzed national training plans.

Until the year 2005, the training in Obstetrics and Gynecology in the Netherlands was semi-structured based on rotations over several clinical departments and a master-apprentice model. The higher numbers of trainees and the increasing complexity of the profession were the main reasons to develop a national training plan with a deliberate structure, both in content and in educational philosophy. This coincided with the advent of CBME and the publication of the first CanMEDS competency framework. The structure and clear outcome description offered by a CBME approach led the curriculum design committee to opt for this approach. The designers realized that although the most up to date educational knowledge was used for the training plans, experience gained by years of practice and developments in educational science, in the field of Obstetrics and Gynecology and in the health systems itself would urge a regular update.

Since the introduction of CBME, there have been 3 consecutive training plans (TP) for obstetrics and gynecology. These plans were published and subsequently implemented in 2005 (TP’ 05), 2013 (TP ‘13), and 2021 (TP’ 21).

TP ‘05 was the first competency-based Dutch national obstetrics and gynecology training plan. This training plan adopted the 1996 competency model of the Canadian Medical Education Directives for Specialists (CanMEDS).

TP ‘13, the second postgraduate training plan, contained a core training plan and newly introduced electives. The core training plan included all the general required learning outcomes of the specialty training and constituted the training program’s core, which was equally compulsory for each resident. By contrast, the electives allowed for additional in-depth training in certain domains of obstetrics and gynecology. In addition, some forms of time-variable learning were implemented, which had become possible due to changes in legislation.

TP ‘21, the most recent training plan, introduced a new concept: in addition to a core training plan with electives, this new plan also featured themes, i.e., descriptions of topics in which residents can develop work-related competencies and pay attention to personal and professional development.

### 2.2 Data analysis

Data were analyzed between January and June 2023. The steps in this process, which are listed in Table 1, were based on the step guide for template analysis of King et al. (31). We started by defining *a priori* themes based on literature: CBME in practice, assessment, and time-based versus time-variable competency-based learning (11, 12). Next, the first author read the training plans, followed by initial coding. The first author shared the outcomes of initial coding with the research team for joint analysis. Based on this analysis, the team developed the template. If codes did not fit the *a priori* themes, we added new themes. Subsequently, the final coding process started, using iterative coding. The research team discussed the definitive template and codes. Data management and coding were performed with Atlas.ti.

**Table 1:** Steps in the analysis.

1. Definition of a priori themes
2. Familiarise oneself with the documents
3. Initial coding
4. Template development and final coding
5. Listing themes
6. Interpretation
7. Quality and reflexivity

### 2.3 Rigor and reflexivity

All the members of the research team are involved in medical education research. The team has expertise in education, medical practice and postgraduate training plan design. MH has clinical experience in obstetrics and gynecology. She performed the coding and used a logbook and reflective memos throughout the entire process of this study.

FS is a gynecologist and a professor of health systems innovation and education in Amsterdam. His area of expertise is health system innovation and education. He was involved in the development of the three training plans, and he is the author of several articles on the effects and difficulties of implementation strategies.

PT, also a gynecologist, has expertise in education development and medical education research and was involved in developing the 2021 training plan. As the chair of the revision committee, he used the growing body of research on work-based education and evaluations of CBME and work-based assessment as an inspiration for the revision of the O&G curriculum. As a researcher in the field, he critically examines how any educational intervention impacts practice and supports or, at times, hinders learning in and from practice. His involvement in the field as a gynecologist, a health professions education researcher and a curriculum designer shaped his interpretations of the data.

ED is an educationalist with expertise in medical education development and research. He has expertise in training plan development in several specialties. Since 2005, he has studied the implementation of CBME and has developed and led workplace-based faculty development sessions for his training region. The faculty development sessions made him aware of the frictions in CBME practice and of how trainees and teachers cope with these frictions. This background shaped ED’s interpretations; he considered how each curriculum incorporated the theoretical developments and the experiences of the trainees and teachers.

## 3 Results

### 3.1 Themes

Our thematic analysis of the differences between the consecutive training plans revealed changes that could be summarized in three themes:

- Theme 1: from a fixed to an open training plan
- Theme 2: increasing degrees of freedom in personalized training trajectories
- Theme 3: assessment, from accountability to trust

The three themes will be described below, and we will use quotes from the three training plans to support the outcomes of the thematic analysis. The three main lessons learned from these themes are collected in Table 2. We also found some recurrent elements that were essential for all three training plans. We will present these recurrent elements at the end of the results section. The results are summarized in Table 3.

**Table 2:** The three main lessons learned.

*From a fixed to an open training plan* 1. In the Dutch context, a structured postgraduate training plan, with elaborate assessment and a fixed outcomes matrix developed towards a partially open training plan. Besides the fixed core of the specialty, there may be given room for both electives and themes with societal relevant themes to choose from. Not all learning outcomes of the specialty training were the same for every resident. *Increasing degrees in freedom in personalized training plans* 2. There was an increase in the degree of freedom and personalization throughout the training plans. The balance shifted from a solid foundation with focus on the intended learning outcomes, towards room for individual development and attention for both professional and personal development. Providing opportunities for transformative learning. *Assessment, from accountability to trust* 3. There can be a distinction in domains that need concrete assessment and domains that can be followed up differently. Parts of the training can also be guided with trust towards the residents, best effort obligation, self-reflection and a feedback culture.

**Table 3:** Summary of results.

	2005	2013	2021
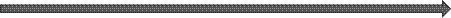			
**Theme 1** From a fixed to an open training plan	Fixed training plan ILOs* same for all	Core training plan and electives ILOs may differ depending on electives, same within elective	Core training plan, electives and themes Personalized ILOs for themes ILOs differ for electives
	Outcome-based education in line with CanMEDS terminology All ILO’s captured in EPA’s	Outcome-based education in line with CanMEDS terminology All ILO’s captured in EPA’s	Growth-and outcome-based education in line with workplace-based education concepts. ILOs covered by a combination of EPA’s and themes.
**Theme 2** Increasing degrees of freedom in personalized training trajectories	Area of interest: Residents could spend additional time in an area of interest, if the set ILO’s were achieved	Choice of elective: 4 general years and 2 years of electives, in those 2 years there was freedom of choice within the elective options Emphasis on personal wellbeing	Choice of elective Freedom within themes: when, how and what within 5 developmental themes with personalized learning outcomes. Attention for professional and personal wellbeing and development
**Theme 3** Assessment, from accountability to trust	Frequent assessment in various, mostly summative forms	Introduction of programmatic assessment combining assessment for formative and summative purposes	Discontinuation of target numbers in assessment Introduction of “best effort obligation” in themes with self-reflection Focus on trust in the learning process of the resident
**Recurrent elements**	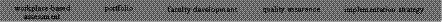		

^*^ILOs: intended learning outcomes.

### 3.2 Theme 1: from a fixed to an open training plan

In this section, we focus on the structure of the training plan. Our analysis showed a development from a training plan in 2005 that was fixed in terms of intended learning outcomes, assessment and competencies, to a training plan in 2021 that is partially open-ended. By a fixed versus an open training plan, we mean the following: in a fixed training plan, all the intended learning outcomes and the ways to reach those outcomes are set and the same for every resident. In an open training plan, not all the intended learning outcomes are fixed, they are not the same for every resident and the ways in which residents reach their end goals can differ as well.

#### 3.2.1 A fixed training plan

TP ‘05 was the first plan that introduced a competency-based model into postgraduate medical education. It incorporated competencies and placed a strong emphasis on assessment and portfolios. The training plan comprised detailed instructions for how the plan should be executed in the training regions and hospitals. All the intended learning outcomes were the same for every resident. Competencies were elaborated into sub-competences and linked to entrustable professional activities (EPAs). The intended learning outcomes combined should result in a good gynecologist.

#### 3.2.2 Keep up with expansion of medical knowledge

TP ‘13 placed greater emphasis on the implementation of competency-based learning and adjusted to changes in the medical field, such as the increasing expansion of medical knowledge. An important element was the introduction of electives in the training plan, in addition to a core training plan. While the core training plan, which covered the basis of the specialty training, was the same for every resident, the electives allowed residents to further deepen their knowledge in certain domains of the specialty (e.g., benign gynecology, oncology, and obstetrics). *“It has been established that developments within the field are happening so quickly that no one is able to keep up with the knowledge explosion that is taking place for all the electives. So there is a certain concentration (on the electives) after the broad foundation has been laid*.” *–* TP ‘13. However, residents’ training plans still had to fit within the clearly defined learning outcomes and assessment framework.

#### 3.2.3 A more open training plan

TP ‘21 introduced a new concept based on an increasing body of research on how aspects of CBME and assessment work in practice. TP ‘21 continued the structure of a core curriculum combined with electives but with the addition of themes. TP ‘21 describes four themes that cover topics in which residents can develop their work-related competencies, knowledge and skills and pay attention to their personal and professional development: work-life balance and work enthusiasm, network health systems, organization of health, knowledge and innovation. *“Ongoing developments require healthcare providers to have an adaptive and creative view that extends beyond the boundaries of their own specialty and the hospital. One which increasingly uses insights from fields such as change management. … It means that it is essential that we learn together to deal with changes and disruptions in a more strategic and educated way.”—TP’21.* This argument was one of the reasons underpinning the decision to create a partially open-ended training plan. The core training plan is still fixed in terms of the intended learning outcomes, but it is up to the residents to determine how they will achieve these outcomes and in which order. The training plan provides even more openness in structure in the electives and themes.

### 3.3 Theme 2: increasing degrees of freedom in personalized training trajectories

This second theme is the counterpart of the first theme, which focused on the changes in the structure of the training plans. With an increasing openness in the structure, the space for personalized learning trajectories increased as well. Each revision provided more freedom of choice regarding the focus of personal and professional development. TP ‘13 and TP ‘21 stipulated that residents should not all follow the same learning path. Although every resident should achieve similar core outcomes, both plans offered room for individualized development beyond the core objectives. Below, we will describe how this personalization has developed and how it was operationalized in the training plans.

#### 3.3.1 Area of interest

TP ‘05 offered little room for personalization. Every resident would follow the same training plan with the same learning outcomes. There were no electives. However, there were some opportunities for personalization in learning trajectories. For example, residents could expand on an area of interest if they achieved the competencies more quickly than their peers. “*Based on the portfolio, the fast resident will be able to demonstrate that he or she has more time for the area of interest.” – TP ‘05*. The area of interest could for example be a specific medical domain within O&G training or specific surgical skills. Furthermore, the training plan described fixed intended learning outcomes, but the resident and the local training institution could decide on the order in which the resident achieved the competencies of those intended learning outcomes.

#### 3.3.2 Electives

TP ‘13 introduced electives in addition to a core training plan. With the increase in knowledge in the medical field and the increasing number of sub-specializations after specialty training, the training plan was adapted to the fact that it was no longer feasible for all residents to achieve the same intended learning outcomes. The former intended learning outcomes were revised and became partially optional in the form of electives. Residents could spend the time left after their core training on the electives of their choice. “*In addition to the core training plan of four years, TP ‘13 introduces a two-year elective period so that every gynecologist is broadly employable but can also be deployed in an area of interest. This has marked the beginning of a new phase” – TP ‘13* As well as providing room for electives, the training plan also offers room for personalization by paying attention to personal wellbeing. The plan indicates that during the annual reviews, program directors should not only evaluate the residents’ professional performance, but also their personal well-being and their work-life balance, since these factors also influence professional functioning.

#### 3.3.3 Development themes and electives

TP ‘21 explicitly describes personalization, in various forms: through electives, themes and attention to personal development. TP ‘21 preserved the electives and the core training plan of TP ‘13 and complemented them with four themes that focus on work-related competencies as well as on personal and professional development: work-life balance and work enthusiasm, network health systems, organization of health, and knowledge and innovation. The themes have no set outcomes; the residents are free to develop, albeit under best effort obligation, which means that they are expected to demonstrate their best efforts and growth within each of the themes. The development is monitored via self-reflection, progress conversations between residents and supervisors, and a portfolio. The authors of TP ‘21 state that developing a variety of skills and interests in combination with the core training plan will result in a good gynecologist: “*TP ‘21 is characterized by the confidence in residents and gynecologists that they carefully train future gynecologists… with the space to realize individualized training trajectories in addition to a solid foundation.” TP ‘21.*

### 3.4 Theme 3: assessment: from accountability to trust

Our analysis of the three training plans showed a clear change in assessment strategy. TP ‘05 and TP ‘13 applied the pedagogical approach “assessment drives learning,” which resulted in an assessment matrix with a high frequency of clinical feedback and skills assessments, clinical evaluations, knowledge exams, simulation exams and progress meetings, which were collected in a portfolio. By contrast, TP’21 applied a more development-oriented assessment approach, based on the assumption that it is not necessary to measure every developmental aspect. Rather than assessing whether a development took place, TP ‘21 introduced a combination of assessment and best effort obligation, with trust in the learning process. *“We do not see a competent gynecologist as the sum of ticked off competences.”* – TP ‘21.

#### 3.4.1 From numbers to narratives

TP ‘05 used frequent assessment in various, mainly summative, forms. “Target numbers can be useful to ensure sufficient experience for the resident. It is not easy to properly assess competency levels… it is safer not to abandon the principle of numbers, which guarantee experience.”—TP ‘05. All the intended learning outcomes of the training plan were translated into EPAs, which were assessed throughout the training plan with various assessment forms and at different levels of independency. Each resident’s progress was documented in a portfolio. It was thought that residents who had demonstrated their capacity to perform the required EPAs in these different forms of assessment would become good gynecologists. “The resident is entitled to sufficient participation in practical situations with professional coaching from the trainer. The trainer expects the resident to demonstrate, with the aid of EPAs and a portfolio, a growth of competences according to plan.”—TP ‘05.

In the subsequent training plans, the assessments increasingly included narrative feedback in combination with attention to personal development. TP ‘13 maintained the assessment forms of TP ‘05 and extended those with narrative feedback options. For example, when trainers assessed an EPA, they could add narrative feedback to the level of independency. The same option was available for the clinical assessment of surgical skills or for the assessment of communication skills. Narrative feedback was collected in the portfolio, as were the other forms of assessment, such as the outcomes of knowledge exams and the independency levels achieved in various EPAs. The narrative feedback was used to monitor progress and could refer to different competency domains.

#### 3.4.2 Towards a feedback culture

TP ‘21 stressed the importance of a feedback culture: “Feedback is one of the most important ways to help a resident learn. In order for a resident to effectively give and receive feedback, a supportive climate and feedback culture are required. This means that it should be considered normal to provide each other with explanations and feedback.” – TP ‘21. The training plan was designed to find a balance between assessment of learning and assessment for learning. The plan placed less emphasis on summative assessment than the previous plans and reduced the frequency of assessment. The role of formative assessment in the form of narrative feedback was more pronounced.

The assessment of EPAs differed from the assessment of themes. EPAs were assessed at different independency levels and with specific criteria, whereas themes were evaluated through self-reflection based on best effort obligation and via narrative feedback in the portfolio. Since best effort obligation meant that there was no concrete assessment on the theme, learning outcomes were no longer all captured in EPAs. TP ‘21 introduced a distinction between criterion-based assessment and learning objectives with demonstrable development. Learning outcomes that need concrete assessment (for example, for the sake of patient safety) were criterion-based. “Some themes are mainly about providing guidance for development in areas for which we cannot establish universal end goals. Feedback on development within the themes will often require openness and self-reflection of the resident and the supervising trainer. To facilitate this conversation and create some form of transparency around the resident’s development within the themes, the residents are asked to accept a best efforts obligation.” TP ‘21.

### 3.5 Recurrent elements

Our data showed certain recurring elements, which were fundamental for the structure of the training plans and were developed alongside the content of the training plan.

#### 3.5.1 Workplace-based assessment and portfolio

All training plans used workplace-based assessment, with a portfolio system to collect feedback and to show the residents’ progress in earning EPAs and their development throughout the specialty training.

#### 3.5.2 Emphasis on faculty development

TP ‘05 was the first training plan for residents that also introduced faculty development. The training plan stipulated that the staff of the hospitals should be trained in CBME through “teach the teacher” training. It also indicated that trainers should be trained in the theory of competency-based learning, in giving feedback, in assessing entrusted professional activities and in the use of the portfolio. The two subsequent training plans also strongly emphasized the importance of faculty development, which was described as a key factor for implementation in practice. *“Teach the teacher-training plans are the fundament for quality improvement of residency training”—TP ‘05.*

#### 3.5.3 Quality assurance

All three training plans pay attention to quality assurance. They state that training regions should meet certain criteria of quality. Training centers are asked to perform their own quality assurance through a PDCA-cycle: plan-do-check-act, which should involve residents, trainers and other relevant stakeholders. TP ‘21 specifies that achieving improvements requires an open feedback culture and willingness to change and refers to the following quote concerning the need for an open feedback culture: *‘Quality is cultivated by people learning together.’ (33)– TP ‘21.*

#### 3.5.4 Implementation strategy

All three training plans present an implementation strategy that indicates how the training plan could best be implemented in training regions and hospitals. The implementation strategies included change management strategies, timelines, and planned visits to the training region’s academic hospital and to the associated general hospitals. The strategies also included evaluations and collecting feedback from the work floor to improve the training plan and to improve the implementation. *“To implement the new training plan, it is not enough to publish this document. In order to succeed, there must be an actual implementation.” TP ‘13.*

## 4 Discussion

In this study, we aimed to describe the development of CBME translations to curriculum design, based on three consecutive postgraduate training plans spanning 17 years. By exploring how CBME has been used in successive O&G training plans, we aimed to elucidate how CBME-based curriculum design has evolved and which lessons curriculum designers have learned.

The way CBME was translated into training plans has evolved in the course of 17 years of experience with CMBE-based education. The main areas of change were the structure of the training plans, which became increasingly open, the degree to which learning outcomes were mandatory or not, and the way these outcomes were assessed. The last training plan introduced certain learning outcomes that were not fixed and that could therefore be adapted very well to the local context and needs of training hospitals, as well as to the personal interests of the residents. These adaptations can take place without distracting from the core of CBME, which is that residents develop the competencies required for their profession while they work in practice. The residents can grow towards the point where they can be entrusted to perform professional activities. In addition, they can also acquire the competencies needed to cope with future developments.

### 4.1 Time-variable learning

While time-variable learning is often presented as one of the core components of CBME (12, 34), recent studies noted that time-variability is in conflict with practice, due to the high burden that can be imposed on the organization and the workforce (24). This burden is described as logistic chaos (2), calling into question how the theory can be reconciled with practice in both the educational frameworks and the existing healthcare systems (20, 24). In our analysis of the three training plans, time-variability did not emerge as a separate theme, which is in contrast to the outcomes of our literature search and our initial template. Instead, an important theme that emerged was room for personalization. Time-variable learning is compatible with a fixed training plan; the intended learning outcomes should be reached during the course of residency, but if they have not been reached at a certain moment, more time can be granted. A partially open training plan makes fully time-variable learning less relevant and provides room for personalization in achieving competence: in addition to the compulsory learning outcomes of the training plan, there are also preference-related learning opportunities. Fully time-variable learning could lead to an erosion of learning opportunities if learners were to move on to new departments once they obtained the compulsory learning targets because in that case, other, non-compulsory learning opportunities could disappear (35).

### 4.2 Placing our lessons in context: trialability, the assessment burden and transformative learning

**Figure 1:**
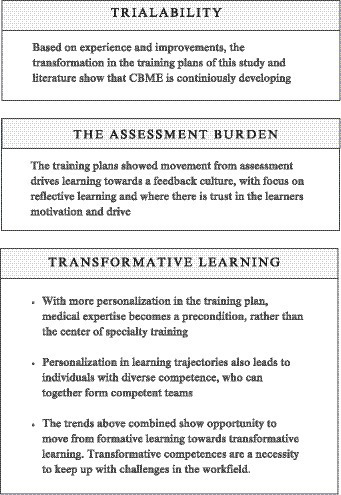
Interpretation and extrapolation of trends.

Our analysis of the differences between the three consecutive training plans demonstrated the advancing insights into the capacity of a CBME-based curriculum to accommodate the challenges of CBME that were described in the introduction: the practicality of CBME (20, 21), the assessment burden (23) and the developments in the work field (18, 24). Figure 1 provides insight in the interpretation and extrapolation of trends of the evolving CBME landscape in postgraduate medical.

#### 4.2.1 The practicality of CBME: trialability

Challenges regarding the practicality of CBME are often debated in terms of a balance between the strict implementation of CBME within a set framework and sufficient flexibility for a local context. Strict implementation is often considered to serve the intended goal of CBME, but a strict framework appears to be not realistic in practice or not in line with implementation literature (7, 21, 28, 29). As a strength, authors state that the potential of CBME lies in its clarity about requirements and in strictness of implementation (36, 37). However, CBME is an innovation that requires involvement and willingness of trainers, legislation adaptations and an augmented workforce (2, 38, 39). While different contexts may require a different balance between strictness and flexibility, our analysis of the way in which the three training plans were redesigned, showed that the concept of CBME is suitable for trialability, i.e., the degree to which an innovation may be experimented with on a limited basis (40, p. 16). According to Rogers, trialability is one of the most important preconditions for successful implementation of innovations. Trialability is positively correlated with the rate of adoption (40). In the implementation stage of the innovation-decision process, reinvention may occur during the trial of the innovation. Then, the innovation may be changed or modified by the potential adopter.

Dagnone et al. stated that CBME should be implemented under tactical guidance from CBME leaders, with committed stakeholder investment at local levels (41). On the one hand, the theory and the experts should be leading the way. However, in practice, there should be room for local diversity, shared leadership, re-invention and development to implement this complex change in medical education (41). Throughout our analysis of the three training plans, trialability was evident, including evaluations from the work floor. The consecutive training plans incorporated lessons learned from practice: maintaining the prior strengths while trying to overcome previous challenges and to implement new insights.

#### 4.2.2 The assessment burden

The assessment of CMBE has been described as a burden on residents and teachers alike. For residents, it constituted a mental and practical burden due to the high frequency of observations and assessments, which teachers experienced as a time-consuming burden because of the frequent moments of direct observation and the cumbersome administration of assessment outcomes (23). A recent scoping review on CBME discourses describes the workload of assessment a challenge as well (8). They added uncertainty of the uniformity of quality of assessment to this conversation. TP ‘21 distinguished between learning outcomes that must be formally assessed (also for the sake of patient safety) and learning outcomes that can be evaluated by other forms of monitoring residents’ progress. In addition to reducing the frequency of observations and assessments, the ‘21 program describes a feedback culture: opening the dialogue between residents and faculty so that they can both learn from each other in a safe learning environment (42). This culture can foster trust in the learning process of the residents, allowing learning outcomes in certain domains to be monitored through reflection and narrative feedback.

#### 4.2.3 The developments in the workfield: transformative learning

The three training plans stressed the importance of incorporating new educational insights, changes in the medical field and innovations in healthcare systems. This finding is in line with international literature (7, 16, 24, 43) and with contemporary educational insights regarding the importance of transformative learning (25). Our analysis showed that opportunities for transformative learning were provided by the partially open training plans of TP ‘13 and TP ‘21 and their increasing emphasis on narrative feedback and self-reflection. Transformative learning is the expansion of consciousness which enables individuals to question their own feelings, beliefs, and assumptions, and their perspective (44). Mezirow (44) and Frenk (43) have shown the importance of transformative learning for critical reflection and for developing new perspectives, which are vital in achieving changes such as innovations in health care systems. A special interest group of the Dutch federation for medical education investigated the modernization of postgraduate medical education and they concluded that transformative learning is still in its early stages in postgraduate medical education, despite its importance in health care transitions (45).

The next step could be to actively implement transformative learning in training plans. The training of professionals and the education of ‘enlightened change agents’ for transformation in health care can become possible with transformative learning, more governance and support from academic leaders with a broader perspective on the future of health care (45). Further research is needed to gather the international perspectives of health professionals and educationalists on healthcare education and to integrate them in the practical implementation of transformative learning.

### 4.3 Limitations and future research

One of the limitations of this study is that it focuses on the CBME training plans developed in a single nation and for a single specialty. In different countries and regions, legislation and health care systems may be different, which may strongly influence the design of training plans. In this study we did not aim to generate generalizable results. However, despite the previously mentioned limitations, the shared experience and the developments in the training plans have potential value for curriculum designers in other medical specialties and in other countries as well. Our findings may transcend our specific research context in both CBME content that is used in similar ways elsewhere, as well as the link to the mentioned challenges, that are not unique to O&G or the Netherlands. Therefore, we expect our results to be helpful to other contexts, for example in the (re) design of a national curriculum.

Future research might analyze training plans designed in a different country or for different specialties. More data should become available on practical experiences and developments over time since CBME is used increasingly around the world and in different specialties. The practical experiences could focus on the outcomes and effects of CBME training plans on learners, patients, and faculty to bridge the gap between theoretical adaptations and real-world impact. We would also recommend investigating the experiences of learners within CBME frameworks, which could contribute to a more holistic understanding of the impact of these educational approaches on medical professionals (in training).

Future research should identify best practices for the implementation of CBME (9). Sharing experiences and lessons learned over time could improve the implementation and the practicality of CBME. We would also recommend researching the outcomes and learning effects of the partially open structure and the assessment approach used in TP ‘21.

In this paper, we did not investigate the enactment of the training plans nor the effects of the three CBME training plans on learners, patients and faculty. A previous study indicated that the effects of CBME on learners have not yet been explored (9). Future research could focus on learner experiences to underpin the theoretical foundation of CBME and to evaluate recent training plan innovations.

## 5 Conclusion

Our analysis of three Dutch training plans showed that the training plan designers chose to re-adjust the training plans with attention to alignment of theory and practice. In doing so, they balanced a solid foundation in a core training plan with a partially open training plan, with room for professional and personal development, expanded with themes that provide opportunities for acquiring competencies beyond the scope of current medical content itself.

To utilize the full potential of CBME while reducing the assessment burden and conflicts with practice, curricula could place greater emphasis on trust towards residents, creating learning opportunities for new competences.

## Data availability statement

The datasets presented in this study can be found in online repositories. The names of the repository/repositories and accession number(s) can be found in the article/supplementary material.

## Author contributions

MH: Conceptualization, Data curation, Formal analysis, Investigation, Methodology, Project administration, Resources, Software, Validation, Visualization, Writing – original draft. ED: Conceptualization, Formal analysis, Methodology, Supervision, Validation, Writing – original draft. PT: Conceptualization, Formal analysis, Methodology, Supervision, Validation, Writing – original draft. FS: Conceptualization, Formal analysis, Methodology, Supervision, Validation, Writing – original draft.
